# Cost-utility analysis of an alcohol policy in Thailand: a case study of a random breath testing intervention

**DOI:** 10.1186/s12913-024-11189-4

**Published:** 2024-06-17

**Authors:** Polathep Vichitkunakorn, Roongnapa Khampang, Pattara Leelahavarong, Jiraluck Nontarak, Sawitri Assanangkornchai

**Affiliations:** 1 Department of Family and Preventive Medicine, Faculty of Medicine, Prince of Songkla University, Songkhla, Thailand; 2 Centre of Alcohol Studies, Thai Health Promotion Foundation, Bangkok, Thailand; 3 Siriraj Health Policy Unit (SiHP), Faculty of Medicine Siriraj Hospital, Mahidol University, Bangkok, Thailand; 4 Department of Epidemiology, Faculty of Public Health, Mahidol University, Bangkok, Thailand; 5 Department of Epidemiology, Faculty of Medicine, Prince of Songkla University, Songkhla, Thailand

**Keywords:** Cost-utility analysis, Random breath testing, Alcohol policy, Road traffic injuries

## Abstract

**Background:**

Road traffic injuries are a major concern worldwide, with Thailand facing high accident mortality rates. Drunk driving is a key factor that requires countermeasures. Random breath testing (RBT) and mass media campaigns recommended by the World Health Organisation intend to deter such behaviour. This study aimed to evaluate the cost-effectiveness of implementing RBT in combination with mass media campaigns in Thailand.

**Methods:**

A Markov simulation model estimated the lifetime cost and health benefits of RBT with mass media campaigns compared to mass media campaigns only. Direct medical and non-medical care costs were evaluated from a societal perspective. The health outcomes were quality-adjusted life years (QALY). Costs and outcomes were discounted by 3% per year. Subgroup analyses were conducted for both sexes, different age groups, and different drinking levels. Probabilistic sensitivity analyses were conducted over 5,000 independent iterations using a predetermined distribution for each parameter.

**Results:**

This study suggested that RBT with mass media campaigns compared with mass media campaigns increases the lifetime cost by 24,486 THB per male binge drinker and 10,475 THB per female binge drinker (1 USD = 35 THB) and results in a QALY gain of 0.43 years per male binge drinker and 0.10 years per female binge drinker. The intervention yielded incremental cost-effectiveness ratios (ICERs) of 57,391 and 103,850 THB per QALY for male and female drinkers, respectively. Moreover, the intervention was cost-effective for all age groups and drinking levels. The intervention yielded the lowest ICER among male-dependent drinkers. Sensitivity analyses showed that at a willingness-to-pay (WTP) threshold of 160,000 per QALY gained, the RBT combined with mass media campaigns had a 99% probability of being optimal for male drinkers, whereas the probability for females was 91%.

**Conclusions:**

RBT and mass media campaigns in Thailand are cost-effective for all ages and drinking levels in both sexes. The intervention yielded the lowest ICER among male-dependent drinkers. Given the current Thai WTP threshold, sensitivity analyses showed that the intervention was more cost-effective for males than females.

**Supplementary information:**

The online version contains supplementary material available at 10.1186/s12913-024-11189-4.

## Background

Road traffic injuries (RTIs) frequently result in death in all age groups and represent a global public health concern affecting everyone who uses roads. Additionally, they place a significant economic burden on society [1]. The incidence of RTIs is higher in low- and middle-income countries than in high-income countries [2].

A 2015 report on road safety in Southeast Asia disclosed that traffic injuries are responsible for 300,000 deaths annually, equating to 17 fatalities per 100,000 people [3]. Among these countries, Thailand had the highest mortality rate owing to RTIs, with 36.2 deaths per 100,000 people, followed by Myanmar with a rate of 20.3. Furthermore, according to a 2011 analysis of the Burden of Disease report regarding the demographics in Thailand, years of life lost (YLLs) owing to RTIs were the second most common cause of death in men, only surpassed by cerebrovascular disease [4]. Apart from vehicle speed, drinking is the most prominent and preventable risk factor associated with RTIs [2]. A noticeable link exists between high blood alcohol levels and the severity of traffic accidents [5].

The globally recognised principle of ‘advance and enforce drink-driving countermeasures’ is a key component of the World Health Organisation’s (WHO) SAFER initiative, which aims to reduce alcohol-related harm [6]. The WHO has identified a set of evidence-based “best buys’ and good buys’ interventions”, that are cost-effective in low- and middle-income countries [7]. The enforcement of drink-driving policies, which can include strategies like random breath testing (RBT), is considered a “good buy” because of its efficacy in reducing alcohol-related accidents, injuries, and fatalities. RBT enhances the perceived risk of detection and potential penalties for drinking and driving and serves as a strong preventive measure [8]. In this practice, police officers randomly stop drivers and compel them to take a preliminary breath test, irrespective of whether they are suspected of wrongdoing. The hallmark of the RBT is its randomness; any driver, at any given time, may be stopped and required to undergo a breath test. To maximise deterrence, RBT is typically performed in a highly visible and extensively publicised manner.

Mass media campaigns act as public information outlets, educating individuals about the risks and penalties associated with drinking and driving and thus attempting to change attitudes and behaviours towards this practice [9]. The impact of these campaigns is greatly enhanced when they are conducted in tandem with RBT, which increases the perceived likelihood of detection and punishment [10]. When combined, RBT and mass media campaigns could have a synergistic effect, strengthening the impact of both interventions [11]. Mass media campaigns can enhance the perceived risk of detection and punishment associated with RBT, thereby augmenting its deterrent effect. In New Zealand, media coverage of the initiative was associated with an additional 14% reduction in similar crashes [12].

Until now, limited research has specifically addressed the cost-utility analysis of combining RBT interventions with mass media campaigns to combat drinking. The effectiveness of these strategies has been explored individually, with studies indicating that both can significantly reduce alcohol-related traffic accidents. However, the cost-utility of these strategies, particularly when implemented together, has not yet been extensively examined. Thus, this study aimed to evaluate the cost-effectiveness of implementing RBT in combination with mass media campaigns in Thailand.

## Methods

### Overview

This study employs a Markov simulation model to project the lifetime costs and health benefits of an alcohol policy, focusing on interventions to reduce RTIs resulting from driving under the influence of alcohol. We used a cohort Markov model adapted from the Scottish Alcohol Intervention Model by Pattara et al. (2018), using epidemiological data available in Thailand [13, 14]. The model simulated multiple conditions, including hospitalisation and death, of alcohol use disorder assessed using the Alcohol Use Disorder Identification Test (AUDIT), and all analyses were classified by sex. Data on the transition probabilities, costs, and health utilities were obtained from the same study. This study considered that short-term outcomes, such as changing risky behaviours and, in particular, drinking under the influence of alcohol, can predict final outcomes such as injury, hospitalisation, and death. From the final outcomes, life years gained, quality-adjusted life years (QALYs) gained, costs incurred from the treatment and rehabilitation services, and the incremental cost-effectiveness ratio (ICER) comparing mass media campaigns only and the RBT together with mass media campaigns were calculated.

### Markov model

The model simulates the health status of individuals who drink alcohol at various risk levels. The population included individuals that had never been admitted to a hospital because of illnesses or health problems related to alcohol use, and they were entered into the model as the study cohort. Subsequently, some of them were hospitalised for various conditions, including (i) wholly alcohol-attributable hospitalisation, (ii) partly alcohol-attributable hospitalisation, and (iii) cardiovascular diseases (CVD) and other conditions, and some died from conditions either related to alcohol or not.

**Fig. 1:**
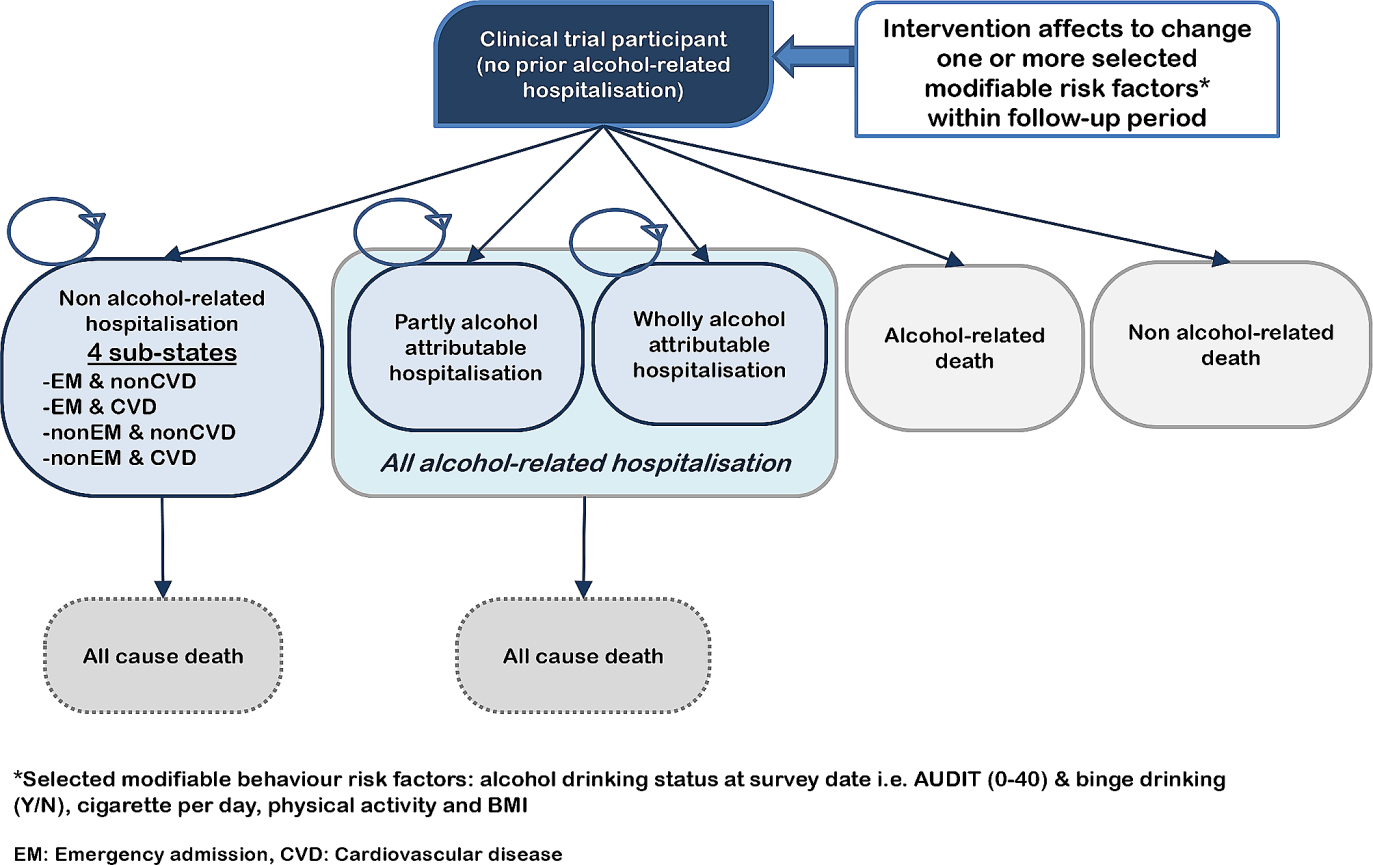
Markov model structure of alcohol intervention. EM: emergency, CVD: cardiovascular disease; *Modifiable risk factors include the AUDIT score (0–40), binge drinking status, number of cigarettes per day, body mass index (BMI), and level of physical activity. The hazard ratio (HR) of each selected risk factor was obtained from the Scottish model [14]

Figure 1 presents a model of the health state of alcohol-related hospitalisation and death in ‘no prior alcohol-related hospitalisation’ cohorts. There are five competing first events classified by the ICD-9 and ICD-10 [15–17] with the primary diagnosis as follows: (1) wholly alcohol-attributable hospitalisation (21 conditions); (2) partly alcohol-attributable hospitalisation; (3) alcohol-related death, which is defined as an alcohol-related hospitalised patient who died within 28 days; (4) non-alcohol-related death, which is defined as a non-alcohol-related hospitalised patient who died within 28 days; and (5) non-alcohol-related hospitalisation, which is divided into four admission types [18, 19]: non-emergency (EM) admission and non-cardiovascular disease (CVD); non-EM admission with CVD; EM admission and non-CVD; and EM admission with CVD.

The selected risk factors used for modelling the first events were age at the survey date, alcohol drinking status at the survey date i.e., AUDIT (0–40) and binge drinking (Y/N), cigarettes per day, CVD condition (Y/N), diabetes (Y/N), physical activity (no activity/low activity/medium activity/high activity), body mass index (BMI) (underweight/normal or BMI < 25, overweight or BMI 25–30, obesity or BMI ≥ 30), socioeconomic status, and subgroup by sex. All statistical analyses were performed using STATA (version 12; StataCorp. LP, College Station, TX, USA).

This study adapted and calibrated the Scottish alcohol policy model using the National Health Examination Survey V [20], the Electricity Generating Authority of Thailand study (EGAT) [21], Thai life tables from the WHO Global Health Observatory data repository [22], and the WHO Global Status Report on Road Safety [23]. The cause-specific hazard for event k at time t is given by Eqs. [24, 25]:

$$h_k (t_i) = \exp (xb) \exp (\gamma t)$$

This equation was then multiplied by a linear predictor using the calibration factor (CF) as follows:

$$h_k (t_i) = [\exp (xb) (CF)] \exp (\gamma t)$$

### Parameters in the model

Direct medical and non-medical care costs were evaluated from a societal perspective. Healthcare costs were obtained from the 2016 Health Administrative Database of the National Health Security Scheme. Annual transition probabilities were adapted from Scottish data adjusted using the Thai National Health Examination Survey, and the probability of the occurrence of CVD was taken from the EGAT study. The utility values for the Thai population were derived from two sources: the Thai National Health Examination Survey (NHES V) and the prevalence of mental disorders and mental health problems (Thai National Mental Health Survey 2013).

### Input parameters specific to interventions to reduce RTIs resulting from driving under the influence of alcohol

Table 1 lists the input parameters used in this analysis. Thailand’s population in 2021 was retrieved from the National Statistical Office [26]. The number of females was higher than that of the male population. The percentage of binge drinkers per total population was obtained from the National Health Examination Survey 2021, showing a higher proportion of male binge drinkers (2.81%) than female binge drinkers (0.92%). We focused our analysis on RBT with mass media campaigns, as evidence suggests its efficacy in the Thai setting. For the comparison, we used mass media campaigns alone because, although attempts have been made to foster the implementation of RBT, such a policy is not currently endorsed in Thailand.

**Table 1:** Input parameters

Parameter	Value	Reference
Population in 2021		
Total	66,171,439	Number in Population from Registration by Age Group, Region and Province: 2012–2021 from the National Statistical Office
Male population	32,339,118	
Female population	33,960,884	
**Percentage of binge drinkers per total population**		
Total	2.4%	Technical paper on patterns and trends of alcohol use behaviour in Thai population, 2022, data from National Health Examination Survey 2021
Male population	2.81%	
Female population	0.92%	
**Number of binge drinkers**		
Total	1,219,987	Calculation
Male population	908,729	
Female population	311,257	
**Efficacy of intervention**		
Random Breath Testing (RBT) with Mass media campaigns (reduced alcohol-related road traffic injuries by 10.6%); efficacy at the current practice where the coverage of sobriety checkpoints was 4.6% and the mass media campaigns coverage was estimated at 100%	10.6%	Ditsuwan et al., 2013
Random Breath Testing (RBT) reduced alcohol-related road traffic fatalities	17.1%	Lacey et al., 1996
**Cost parameters**		
Unit cost of sobriety checkpoints (Selective Breath Testing or RBT) for one case in 2020 adjusted with consumer price index in 2021 Thai baht (THB)	1,322	Royal Thai Police, 2020
Cost of mass media campaigns per year in 2004 adjusted with consumer price index in 2021 (THB)	194,821,538	Ditsuwan et al., 2013
Number of licences in 2018	37,338,139	Global Status Report on Road Safety 2018
Proportion of drunk driving in Thailand	49.8%	The 2021 Health Behaviour of Population Survey
Number of people who benefited from mass media campaign intervention	18,594,393	Calculation from number of licenses in 2018 (37,338,139) x Proportion of drunk driving in Thailand (49.8%)
Cost of mass media campaign intervention per case (THB)	10.48	Calculation from total cost of mass media campaign divided by number of people who benefited from mass media campaign intervention.

Ditsuwan et al. (2013) [27] revealed that if the coverage of sobriety checkpoints was 4.6% and the mass media campaign coverage was estimated at 100%, this intervention reduced RTIs by 10.6%. The RBT intervention, however, reduced drunk driving fatalities by 17%, as reported in a study in the United States of America [28]. We applied these reductions to the Markov model.

The cost parameters associated with implementing sobriety checkpoints were retrieved from a unit cost report of the Royal Thai Police in the fiscal year 2020 and adjusted to the present value using the consumer price index [29]. The sobriety checkpoint’s unit cost was determined by analysing three cost components: labour, materials, and capital. Labour expenses cover salaries, wages, incentives, and fringe benefits for staff involved in checkpoint activities, calculated based on their tasks and time allocation, including vehicle stops, breath testing, and reporting. Materials costs comprised expenses for the breath-alcohol analyser, its calibration and maintenance, training, office supplies, fuel, and utilities. Capital costs included expenditures for building, equipment, vehicles, as well as depreciation and amortisation. The reported unit cost was 1,322 THB (equivalent to 1 USD = 35 THB).

The expenditure on mass media campaigns was reviewed from the study by Ditsuwan et al. in 2013 [27] and adjusted to the present values using the consumer price index. The target population of the mass media campaign was determined by multiplying the number of licenses in 2018, which stood at 37,338,139 according to the WHO Global Status Report on Road Safety 2018 [3], by the proportion of drunk driving in Thailand, estimated at 49.8% from the 2017 Food consumption Behaviour Survey [30]. The cost of the mass media campaign intervention per case (THB) was then derived by dividing the total mass media campaign cost by the number of people who benefited from the intervention. As a result, the expenditure on mass media campaigns was 10.48 THB for one drinker. The sex-specific parameters determined in the model are presented in Table 2.

**Table 2:** Parameters in the base scenario for male and female drinkers

Parameters	Male drinker		Female drinker	
	Mass media alone	RBT and Mass media	Mass media alone	RBT and Mass media
Age	20	20	20	20
AUDIT score	7	7	7	7
Binge	1	0	1	0
BMI	0	0	0	0
CVD	0	0	0	0
Diabetes	0	0	0	0
CPD	4	4	4	4
PhysiAct	2	2	2	2
SES	3	3	3	3
PriorHos	0	0	0	0
GHQ	1	1	1	1

AUDIT: Alcohol Use Disorders Identification Test (score 0–40); BMI: Body Mass Index (0 = Normal, 1 = Overweight, 2 = Obesity); CVD: Cardiovascular Disease condition; CPD: Number of cigarettes per day; PhysiAct: Physical activity (0 = No activity, 1 = Low, 2 = Med, 3 = High); SES: Socioeconomic status (1 = Most Deprived, 2 = 2nd, 3 = 3rd, 4 = 4th, 5 = Least Deprived); PriorHos: Prior Hospitalisation (0 = No PriorHos, 1 = LastYr, 2 = Over last year); GHQ: General Health Questionnaire (1 = Best, 2 = score 1–3, 3 = score 4+). RBT: Random Breath Testing.

Table 2 lists the input parameters indicating the drinking risk profile in the base-case analysis. The age of the base-case group was 20 years for male and female drinkers. The audit score was 7 for both groups, suggesting low-risk consumption. The cohort had a normal BMI, no cardiovascular disease, no diabetes, no prior hospitalisation, the best general health, a medium level of physical activity, smoked four cigarettes per day, and was at the middle level of estimated deprivation.

The health outcomes were QALYs. Costs and outcomes were discounted by 3% per year. The ICER was calculated by dividing the incremental costs of implementing RBT with mass media campaigns by the incremental benefits of implementing RBT with mass media campaigns. The ICERs were presented from a societal perspective. An intervention was considered cost-effective if its ICER was equal to or below the context-specific cost-effectiveness threshold of 160,000 THB per QALY.

### Sensitivity analysis

Subgroup analyses were conducted for the different age groups and drinking levels. In addition, we explored how different assumptions and parameter uncertainties affect conclusions regarding the cost-effectiveness of our intervention by conducting a probabilistic sensitivity analysis. All parameters were varied simultaneously over 5,000 independent iterations using a predetermined distribution for each parameter, as shown in **Appendices 1 and 2**. The results of the simulations are presented using a cost-effectiveness plane and a cost-effectiveness acceptability curve.

## Results

### Base case

This study suggested that RBT with a mass media campaign on drinking and driving compared with a mass media campaign only increases the lifetime cost of 24,486 THB per male binge drinker and 10,475 THB per female binge drinker and would result in a QALY gain of 0.43 years per male binge drinker and 0.10 years per female binge drinker. For the total number of binge drinkers (908,729 and 311,257 for male and female binge drinkers, respectively), the RBT and mass media campaign yielded a total life-year savings of 1,298,919 years for males and 181,143 years for females, as well as a total QALY gain of 387,721 years and 31,395 years for males and females, respectively **(**Table 3**).** When compared with the cost-effectiveness threshold of 160,000 THB per QALY gained, RBT combined with a mass media campaign is cost-effective for male and female drinkers, with ICERs of 57,391 and 103,850 THB per QALY gained for male and female drinkers, respectively.

**Table 3:** Results for male and female drinkers

	Male drinkers			Female drinkers		
	Mass media campaign	RBT with mass media campaign	Incremental	Mass media campaign	RBT with mass media campaign	Incremental
Life years	73.04	74.47	1.43	77.30	77.88	0.58
Discounted life years	44.66	45.16	0.50	44.20	44.36	0.16
Discounted QALYs	41.99	42.42	0.43	33.48	33.58	0.10
Lifetime costs	692,780	739,615	46,835	713,890	731,303	17,413
Discounted lifetime costs	274,286	298,772	24,486	276,917	287,392	10,475
Incremental cost-effectiveness ratio	57,391			103,850		

RBT: Random Breath Testing; QALY: Quality-Adjusted Life Years.

### Subgroup analyses

The subgroup analyses for different age groups among female and male binge drinkers (see **appendix**) revealed that RBT combined with mass media campaigns remained cost-effective for all age groups. The ICERs of the older age groups were lower than those of the younger age groups. Among participants who drank alcohol at various risk levels, we found similar patterns, suggesting that RBT combined with mass media campaigns is cost-effective at all drinking levels. The ICERS score for dependent drinkers was lower than that for drinkers in the moderate drinking group.

### Probabilistic sensitivity analysis

**Fig. 2:**
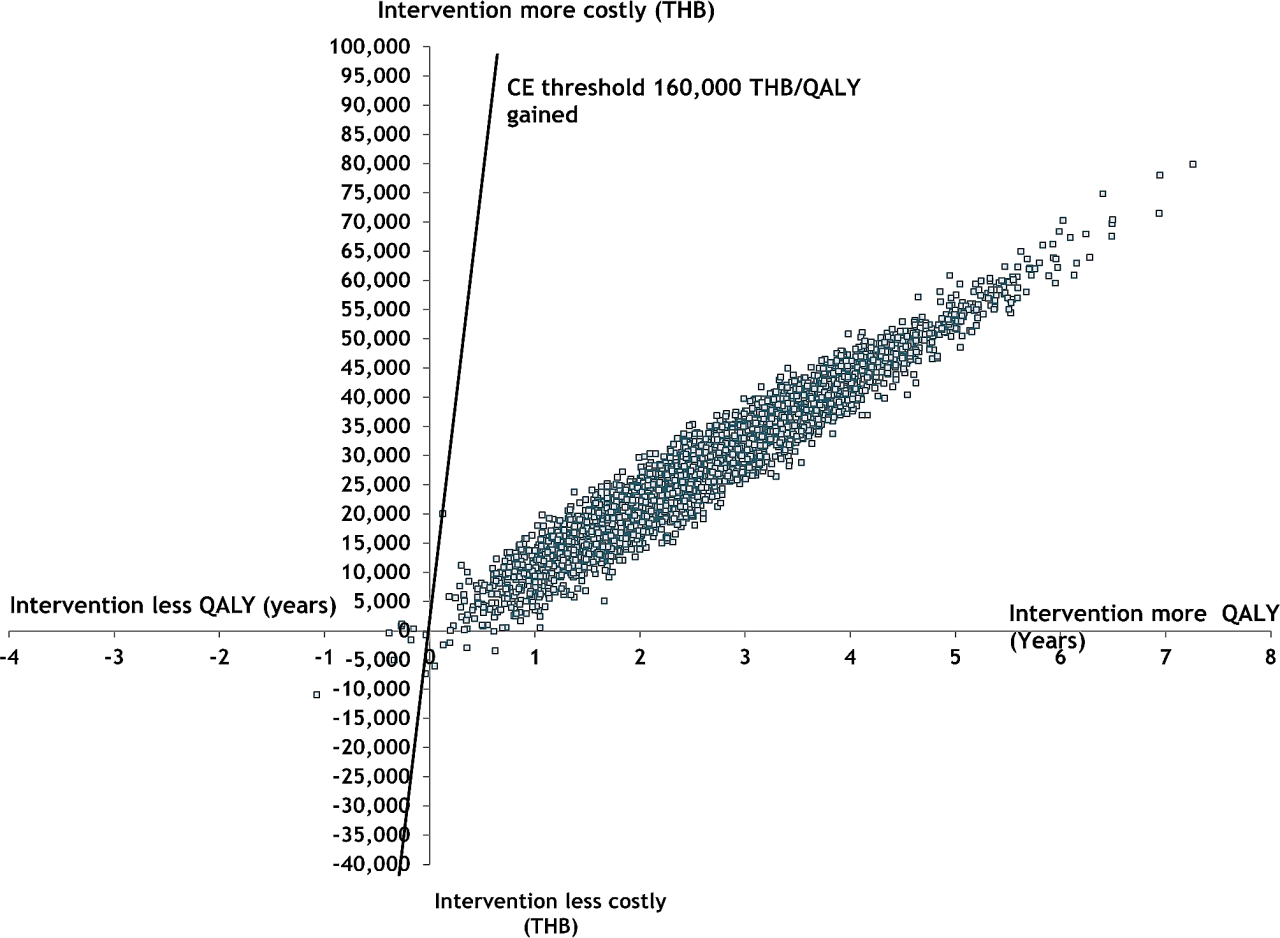
Probabilistic sensitivity analysis on a cost-effectiveness plane for male drinkers aged 20 years

**Fig. 3:**
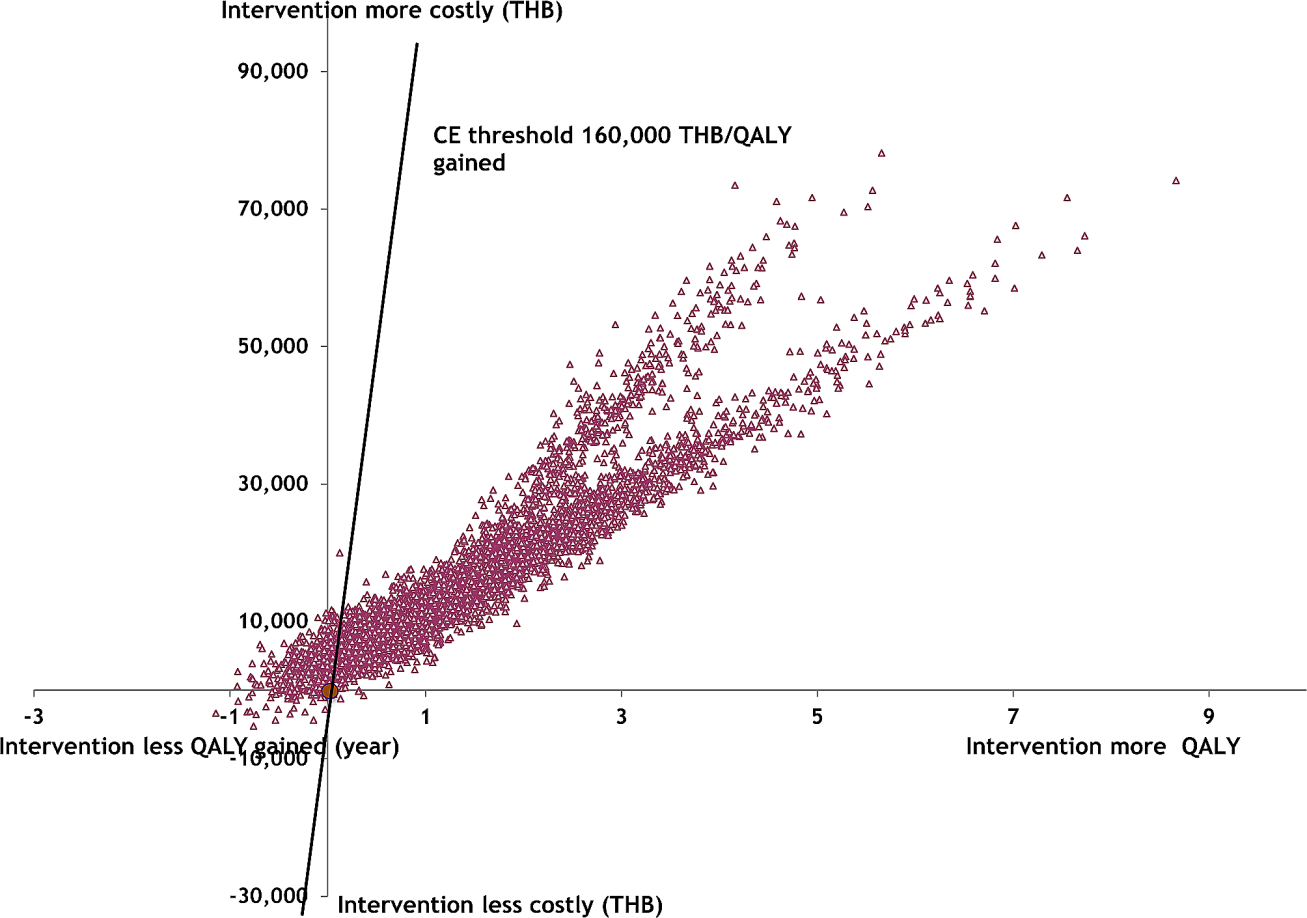
Probabilistic sensitivity analysis on a cost-effectiveness plane for female drinkers aged 20 years

The results of the probabilistic sensitivity analysis are shown in Figs. 2 and 3. The cost-effectiveness planes plot the incremental effectiveness of the intervention against the incremental cost. Each dot in the plot represents the effectiveness and cost of each iteration. The diagonal line in the plot represents the willingness-to-pay (WTP) threshold of 160,000 THB per QALY gained. The cost-effectiveness plots for male drinkers aged 20 years showed that most of the plots were under the diagonal line, suggesting that the probability of the intervention being cost-effective was high. For female drinkers, around 80% of the plots were under the diagonal lines, indicating that the probability of the intervention being cost-effective was also high.

**Fig. 4:**
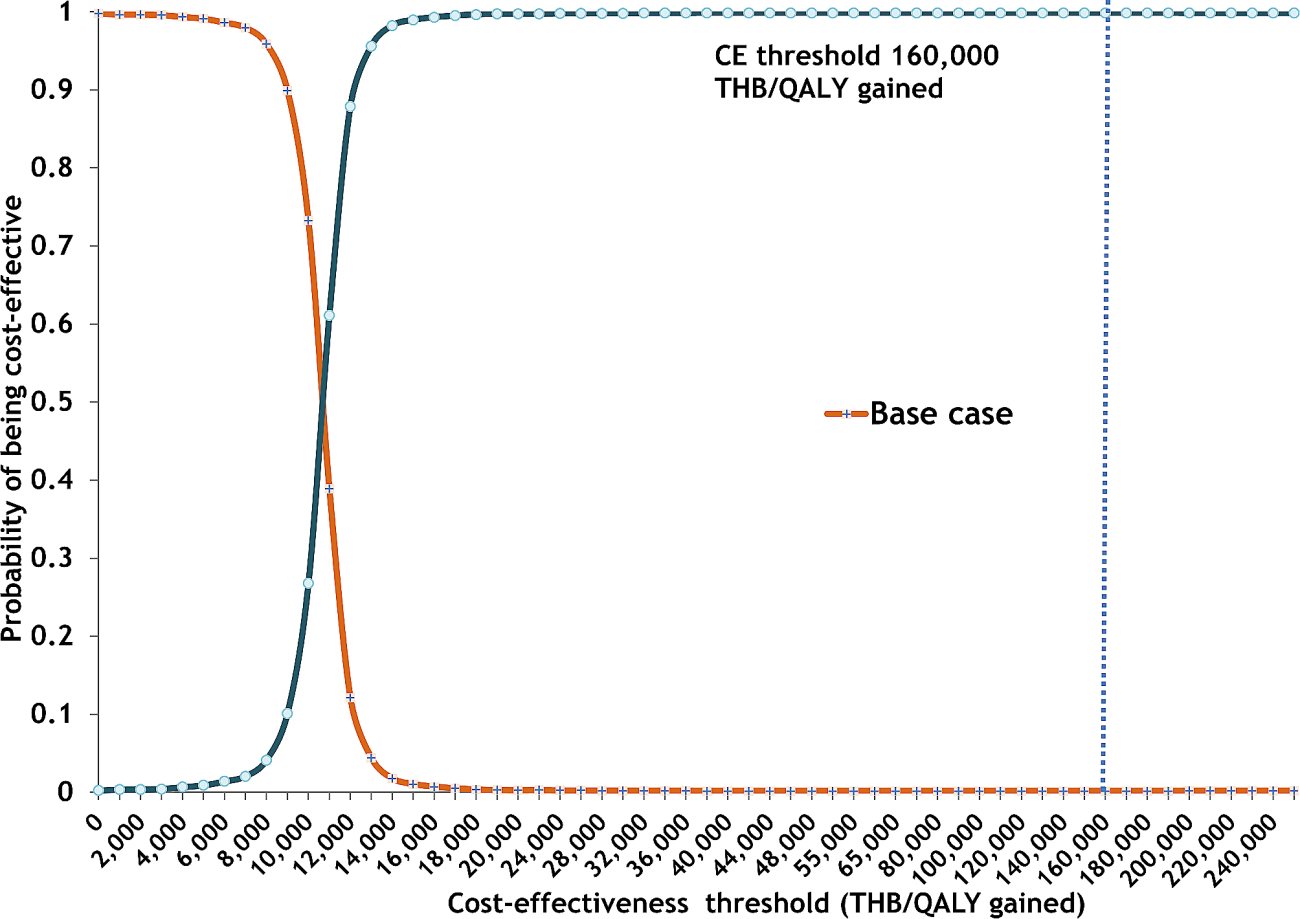
Acceptability curve for male drinkers aged 20 years

**Fig. 5:**
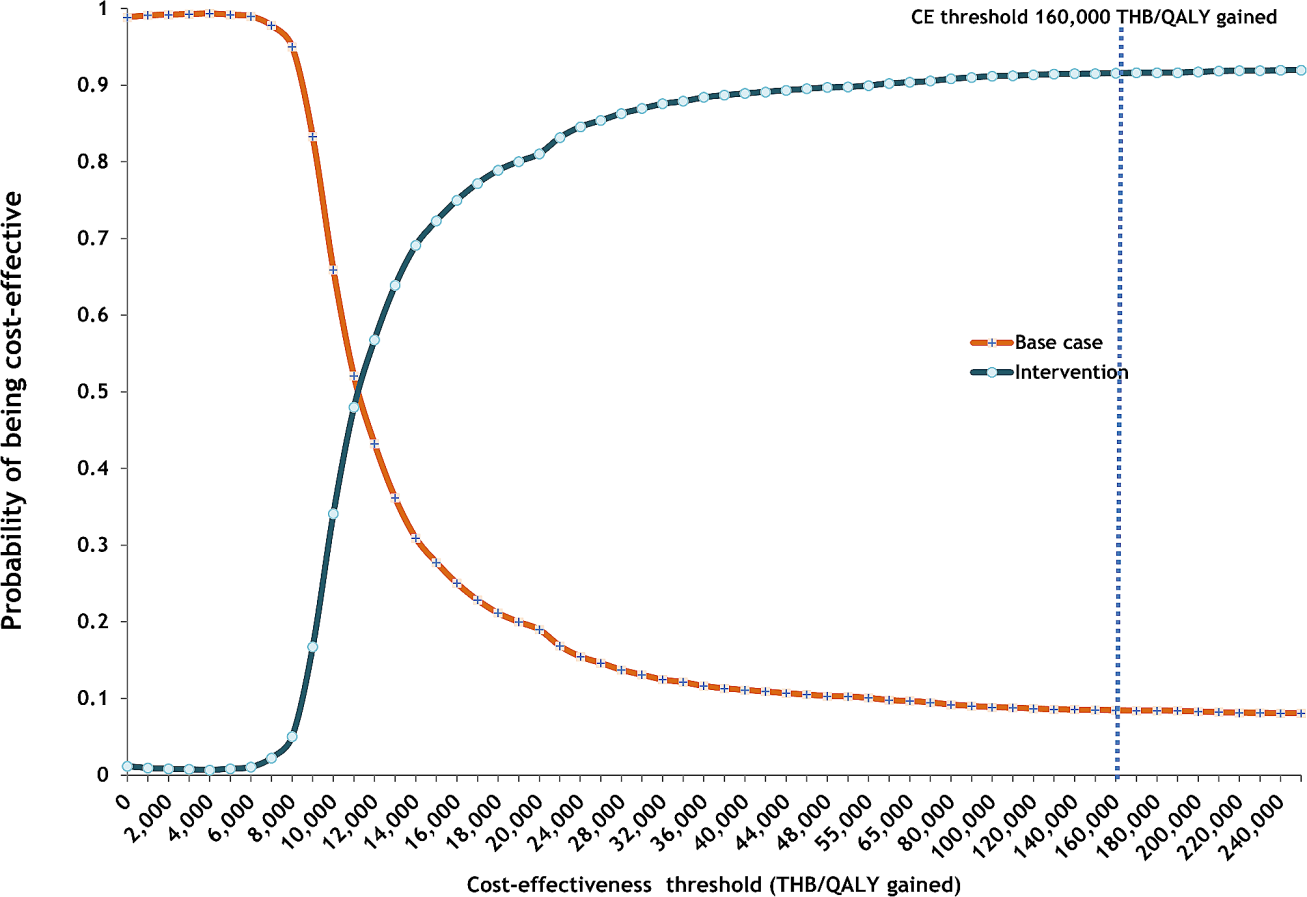
Acceptability curve for female drinkers aged 20 years

When considering the cost-effectiveness-acceptability curve for male drinkers (Fig. 4), our analysis suggests that at any WTP threshold above 10,000 THB per QALY gained, RBT combined with mass media campaigns is more likely to be optimal than mass media campaigns alone. At a WTP threshold of 160,000 per QALY gained, RBT combined with mass media campaigns had a 99% probability of being optimal, whereas mass media campaigns only have a 1% chance. For female drinkers (Fig. 5), the intervention became optimal when the WTP threshold was above 12,000 THB per QALY gained. At a WTP threshold of 160,000 per QALY gained, RBT combined with mass media campaigns had a 91% probability of being optimal, whereas mass media campaigns only had a 9% chance.

## Discussion

### Principal findings and previous studies

This study suggested that RBT with mass media campaigns was a cost-effective intervention for male and female binge drinkers compared with mass media campaigns alone in the Thai cultural context. The intervention yielded ICERs of 57,391 and 103,850 THB per QALY for male and female drinkers, respectively. Moreover, the intervention was cost-effective for all age groups and drinking levels and yielded the lowest ICER among male-dependent drinkers.

The findings of the present study are consistent with those of a 2004 cost-benefit analysis of compulsory breath testing in New Zealand conducted by Miller et al. [12]. It was observed that the compulsory RBT approach yielded greater societal benefit-cost ratios when implemented in conjunction with an augmented mass media campaign. Furthermore, the government realised cost savings owing to a 50% reduction in late-night serious and fatal injury crashes, resulting in societal monetary returns exceeding the costs incurred by implementing the programme.

The cost savings of breath testing were further substantiated in a 2018 study conducted in Australia [31]. This study posited that a 10% increase in breath testing would be linked to a reduction in 0.34 alcohol-related traffic accidents, translating to a statewide decrease of six crashes per month. This programme’s efficacy demonstrates the potential for substantial economic savings for the government and society.

Our study revealed that the majority of QALY gained was attributable to the prevention of binge drinkers driving under the influence of alcohol, resulting from the integration of sobriety checkpoints with mass media campaigns. This observed benefit is largely owing to the relatively low coverage of sobriety checkpoints, currently standing at 4.6%. Consequently, an expansion in checkpoint coverage would yield greater societal returns, highlighting the importance of encompassing all age groups and sexes in the programme. Additionally, our findings indicate that mass media campaigns alone were insufficient in addressing drunk driving behaviours effectively. Therefore, it is critical to expand investments in deploying sobriety checkpoints and intensify law enforcement actions following positive breath test results.

Based on these findings, it is evident that the economic returns and cost-effectiveness of RBT are noteworthy. However, when formulating policy recommendations, a country must consider various factors beyond economic impact. These factors encompass the budgetary implications associated with programme implementation and considerations pertaining to the readiness of the system, community acceptability, and the extent of law enforcement coverage. In the context of low and middle-income countries with limited staff and resources, it is imperative to identify an optimal model for implementing sobriety checkpoints with shorter operation times and fewer personnel.

### Limitations and strengths

This study has certain limitations. First, the effectiveness of RBT in Thailand is inadequately supported by robust clinical trial data. Our analysis relied on a 2013 study that used evidence from a single trial conducted in a single province in a rural area to assess the efficacy and coverage of RBT. The effectiveness of sobriety checkpoints can be influenced by several factors, including their frequency, coverage, and the level of driver cooperation. Urban areas tend to have the capacity to deploy more frequent checkpoints and cover a larger population compared to rural areas. This is owing to the presence of law enforcement resources and breath-alcohol analysers. Furthermore, urban drivers are often more aware of these measures and may be more willing to cooperate compared to their rural counterparts. Therefore, our analysis may underestimate the true effectiveness of the intervention. Second, despite adopting a societal perspective, this model predominantly considers healthcare costs associated with injuries and fatalities as principal expenditures. Owing to data limitations, this investigation was unable to integrate additional expenses linked to road traffic accidents, such as property damage, legal and court fees, productivity losses, and workplace losses. The inclusion of these costs enhances the cost-effectiveness of the interventions.

Nevertheless, this study has several strengths. First, a comprehensive decision modelling study employing the most up-to-date data from Thailand was conducted, yielding cost-effectiveness ratios for various demographic groups of male and female drinkers, considering distinct age groups, risk profiles, and alcohol consumption risks. The model was customised to cater to the requirements of decision-making in specific subpopulations or situational contexts. Second, the input parameters are sourced from multiple local references and validated by critical stakeholders.

### Policy implication

These findings have important policy implications for public health. First, policymakers should be encouraged to support the widespread implementation of RBT paired with mass media campaigns. This approach offers a cost-effective means of enhancing public health and quality of life by curbing excessive alcohol consumption. Second, resource allocation should prioritise comprehensive mass media campaigns that complement RBT efforts. These campaigns can effectively raise awareness about the risks of drinking and promote the importance of alcohol consumption.

Subgroup analyses underscored the universality of the strategy across different age groups and drinking levels for both sexes. This highlights the need for tailored messaging in mass media campaigns to effectively address specific demographics. Regular evaluation is emphasised to ensure that resources are optimally utilised, and the impact of the strategy on reducing alcohol-related incidents is tracked.

Collaborative efforts among various stakeholders, including government bodies, healthcare organisations, law enforcement agencies, and NGOs, are deemed essential because of the multifaceted nature of the issue. Partnerships can facilitate the sharing of resources and expertise, leading to a more comprehensive implementation of RBT and mass media campaign strategies.

In conclusion, this study established that the integration of RBT and mass media campaigns is a financially efficient approach to addressing alcohol-related challenges in Thailand. Evidence of economic viability coupled with the strategy’s adaptability across demographics underscores its potential to enhance public health, safety, and well-being. Policymakers should consider these findings while designing effective interventions and collaborations to combat alcohol-related issues.

### Further studies

Further research is crucial to enhance our understanding of the effectiveness of a combined RBT and mass media campaign strategy in addressing alcohol-related concerns in Thailand. Longitudinal studies could reveal whether the positive effects of the strategy persist over time, indicating a lasting behavioural change. Investigating behavioural change dynamics is vital to grasping how attitudes, beliefs, and societal norms evolve in response to interventions and guiding the refinement of strategies.

A comprehensive cost-benefit analysis should be conducted, encompassing not only healthcare costs but also broader societal benefits such as productivity gains and reduced accident rates. Comparative studies could provide insights into the strategy’s relative effectiveness compared to alternative interventions, aiding resource allocation decisions. Tailoring interventions based on subgroup-specific insights gained through qualitative research can enhance their effects.

Leveraging technology for real-time information dissemination and behaviour tracking can amplify the reach and effectiveness of a strategy. Assessing the strategy’s impact on marginalised groups is essential to ensuring equitable benefit distribution. Multicountry studies can determine a strategy’s cross-context applicability and potential contextual variations. Finally, involving stakeholders from diverse sectors in the research process ensures pragmatic interventions that effectively address real-world challenges.

## Conclusions

The combination of RBT and mass media campaigns proved to be economically efficient in Thailand (with an ICER of 57,391 THB for males and 103,850 THB for females per QALY gained). It is noteworthy that the cost-effectiveness threshold in Thailand is 160,000 THB per QALY gained (1 USD = 35 THB). Conducting subgroup analyses further underscored the cost-effectiveness of the approach, as it remains viable across all age groups and drinking levels in both sexes.

### Electronic supplementary material

Below is the link to the electronic supplementary material.

Supplementary Material 1

## Data Availability

The data sets used and analysed during this study are available from the corresponding author upon reasonable request.

## Declarations

### Abbreviations

AUDIT Alcohol Use Disorder Identification Test.

BMI Body mass index.

CF Calibration factor.

CPD Number of cigarettes per day.

CVD Cardiovascular disease.

DALY Disability-Adjusted Life Year.

EGAT Electricity Generating Authority of Thailand.

HR Hazard ratio.

ICER Incremental cost-effectiveness ratio.

QALY Quality-adjusted life year.

RBT Random breath testing.

RGNS Research Grant for New Scholar.

RTI Road traffic injuries.

SiHP SI Health Policy.

THB Thai Baht.

USD United States Dollar.

WHO World Health Organisation’s.

WTP Willingness-to-pay.

YLL Years of life lost.
