# Supplementary material for: Cost-utility analysis of an alcohol policy in Thailand: a case study of a random breath testing intervention
Source: BMC Health Serv Res. 2024 Jun 17;24:739. doi: 10.1186/s12913-024-11189-4 (PMC11181527; doi:10.1186/s12913-024-11189-4)
Supplement: Supplementary file 1 — Supplementary Material 1 [file 12913_2024_11189_MOESM1_ESM.docx]

# Appendix 1: Sub-group analyses for male drinkers

1. Male drinkers aged 30 years old with low levels of alcohol consumption.

| **Characteristics** | **Mass media** | **RBT+Mass media** |
| --- | --- | --- |
| Age | 30 | 30 |
| AUDIT | 7 | 7 |
| Binge | 1 | 0 |
| BMI | 0 | 0 |
| CVD_con | 0 | 0 |
| Diabetes | 0 | 0 |
| CPD | 4 | 4 |
| PhysiAct | 2 | 2 |
| SES | 3 | 3 |
| PriorHos | 0 | 0 |
| GHQ | 1 | 1 |
| LY | 72.14 | 73.25 |
| Disc LY | 52.08 | 52.54 |
| Disc QALYs | 48.43 | 48.83 |
| Lifetime costs | 570,193.04 | 607,264.14 |
| Disc lifetime costs | 263,415.16 | 284,815.46 |
| ICER per drinker |  | 53,946 |

AUDIT: Alcohol Use Disorders Identification Test (score 0–40); BMI: Body Mass Index (0=Normal, 1=Overweight, 2=Obesity); CVD: Cardiovascular Disease; CPD: Number of cigarette per day; PhysiAct: Physical activity (0=No activity, 1=Low, 2=Med, 3=High); SES: Socioeconomic status (1=Most Deprived, 2=2nd, 3=3rd, 4=4th, 5=Least Deprived); PriorHos: Prior Hospitalization (0=No PriorHos, 1=Last Yr, 2=Over last year); GHQ: General Health Questionnaire (1=Best, 2=score 1–3, 3=score 4+). RBT: Random Breath Testing.

1. Male drinkers aged 40 years with low alcohol consumption.

|  | Mass media | RBT+Mass media |
| --- | --- | --- |
| Age | 40 | 40 |
| AUDIT | 7 | 7 |
| Binge | 1 | 0 |
| BMI | 0 | 0 |
| CVD_con | 0 | 0 |
| Diabetes | 0 | 0 |
| CPD | 4 | 4 |
| PhysiAct | 2 | 2 |
| SES | 3 | 3 |
| PriorHos | 0 | 0 |
| GHQ | 1 | 1 |
| LY | 72.23 | 73.07 |
| Disc LY | 59.03 | 59.45 |
| QALY | 55.01 | 55.36 |
| Disc QALYs | 450,409.05 | 478,781.10 |
| Lifetime costs | 241,762.57 | 259,807.07 |
| ICER per drinker |  | 51,159 |

AUDIT: Alcohol Use Disorders Identification Test (score 0–40); BMI: Body Mass Index (0=Normal, 1=Overweight, 2=Obesity); CVD: Cardiovascular Disease; CPD: Number of cigarette per day; PhysiAct: Physical activity (0=No activity, 1=Low, 2=Med, 3=High); SES: Socioeconomic status (1=Most Deprived, 2=2nd, 3=3rd, 4=4th, 5=Least Deprived); PriorHos: Prior Hospitalization (0=No PriorHos, 1=Last Yr, 2=Over last year); GHQ: General Health Questionnaire (1=Best, 2=score 1–3, 3=score 4+). RBT: Random Breath Testing.

1. Male drinkers aged 50 years with low alcohol consumption.

|  | Mass media | RBT+Mass media |
| --- | --- | --- |
| Age | 50 | 50 |
| AUDIT | 7 | 7 |
| Binge | 1 | 0 |
| BMI | 0 | 0 |
| CVD_con | 0 | 0 |
| Diabetes | 0 | 0 |
| CPD | 4 | 4 |
| PhysiAct | 2 | 2 |
| SES | 3 | 3 |
| PriorHos | 0 | 0 |
| GHQ | 1 | 1 |
| LY | 73.68 | 74.28 |
| Disc LY | 65.68 | 66.02 |
| Disc QALYs | 60.81 | 61.10 |
| Lifetime costs | 341,621.50 | 362,326.66 |
| Disc lifetime costs | 211,148.63 | 225,628.03 |
| ICER per drinker | | 50,271 |

AUDIT: Alcohol Use Disorders Identification Test (score 0–40); BMI: Body Mass Index (0=Normal, 1=Overweight, 2=Obesity); CVD: Cardiovascular Disease; CPD: Number of cigarette per day; PhysiAct: Physical activity (0=No activity, 1=Low, 2=Med, 3=High); SES: Socioeconomic status (1=Most Deprived, 2=2nd, 3=3rd, 4=4th, 5=Least Deprived); PriorHos: Prior Hospitalization (0=No PriorHos, 1=Last Yr, 2=Over last year); GHQ: General Health Questionnaire (1=Best, 2=score 1–3, 3=score 4+). RBT: Random Breath Testing.

1. Male drinkers aged 20 years with hazardous alcohol consumption.

|  | Mass media | RBT+Mass media |
| --- | --- | --- |
| Age | 20 | 20 |
| AUDIT | 15 | 15 |
| Binge | 1 | 0 |
| BMI | 0 | 0 |
| CVD_con | 0 | 0 |
| Diabetes | 0 | 0 |
| CPD | 4 | 4 |
| PhysiAct | 2 | 2 |
| SES | 3 | 3 |
| PriorHos | 0 | 0 |
| GHQ | 1 | 1 |
| LY | 72.24 | 73.93 |
| Disc LY | 44.30 | 44.92 |
| Disc QALYs | 40.26 | 40.77 |
| Lifetime costs | 689,741.45 | 738,700.73 |
| Disc lifetime costs | 275,203.25 | 300,128.05 |
| ICER per drinker |  | 49,162 |

AUDIT: Alcohol Use Disorders Identification Test (score 0–40); BMI: Body Mass Index (0=Normal, 1=Overweight, 2=Obesity); CVD: Cardiovascular Disease; CPD: Number of cigarette per day; PhysiAct: Physical activity (0=No activity, 1=Low, 2=Med, 3=High); SES: Socioeconomic status (1=Most Deprived, 2=2nd, 3=3rd, 4=4th, 5=Least Deprived); PriorHos: Prior Hospitalization (0=No PriorHos, 1=Last Yr, 2=Over last year); GHQ: General Health Questionnaire (1=Best, 2=score 1–3, 3=score 4+). RBT: Random Breath Testing.

1. Male drinkers aged 30 years with hazardous alcohol consumption.

|  | Mass media | RBT+Mass media |
| --- | --- | --- |
| Age | 30 | 30 |
| AUDIT | 15 | 15 |
| Binge | 1 | 0 |
| BMI | 0 | 0 |
| CVD_con | 0 | 0 |
| Diabetes | 0 | 0 |
| CPD | 4 | 4 |
| PhysiAct | 2 | 2 |
| SES | 3 | 3 |
| PriorHos | 0 | 0 |
| GHQ | 1 | 1 |
| LY | 71.43 | 72.77 |
| Disc LY | 51.73 | 52.31 |
| Disc QALYs | 46.47 | 46.95 |
| Lifetime costs | 566,619.33 | 605,482.96 |
| Disc lifetime costs | 263,621.14 | 285,496.50 |
| ICER per drinker | | 45,666 |

AUDIT: Alcohol Use Disorders Identification Test (score 0–40); BMI: Body Mass Index (0=Normal, 1=Overweight, 2=Obesity); CVD: Cardiovascular Disease; CPD: Number of cigarette per day; PhysiAct: Physical activity (0=No activity, 1=Low, 2=Med, 3=High); SES: Socioeconomic status (1=Most Deprived, 2=2nd, 3=3rd, 4=4th, 5=Least Deprived); PriorHos: Prior Hospitalization (0=No PriorHos, 1=Last Yr, 2=Over last year); GHQ: General Health Questionnaire (1=Best, 2=score 1–3, 3=score 4+). RBT: Random Breath Testing.

1. Male drinkers aged 40 years with hazardous alcohol consumption.

|  | Mass media | RBT+Mass media |
| --- | --- | --- |
| Age | 40 | 40 |
| AUDIT | 15 | 15 |
| Binge | 1 | 0 |
| BMI | 0 | 0 |
| CVD_con | 0 | 0 |
| Diabetes | 0 | 0 |
| CPD | 4 | 4 |
| PhysiAct | 2 | 2 |
| SES | 3 | 3 |
| PriorHos | 0 | 0 |
| GHQ | 1 | 1 |
| LY | 71.61 | 72.63 |
| Disc LY | 58.69 | 59.21 |
| Disc QALYs | 52.84 | 53.26 |
| Lifetime costs | 446,826.44 | 476,567.55 |
| Disc lifetime costs | 241,439.68 | 259,930.21 |
| ICER per drinker | | 43,711 |

AUDIT: Alcohol Use Disorders Identification Test (score 0–40); BMI: Body Mass Index (0=Normal, 1=Overweight, 2=Obesity); CVD: Cardiovascular Disease; CPD: Number of cigarette per day; PhysiAct: Physical activity (0=No activity, 1=Low, 2=Med, 3=High); SES

Socioeconomic status (1=Most Deprived, 2=2nd, 3=3rd, 4=4th, 5=Least Deprived); PriorHos: Prior Hospitalization (0=No PriorHos, 1=Last Yr, 2=Over last year); GHQ: General Health Questionnaire (1=Best, 2=score 1–3, 3=score 4+). RBT: Random Breath Testing.

1. Male drinkers aged 50 years with hazardous alcohol consumption.

|  | Mass media | RBT+Mass media |
| --- | --- | --- |
| Age | 50 | 50 |
| AUDIT | 15 | 15 |
| Binge | 1 | 0 |
| BMI | 0 | 0 |
| CVD_con | 0 | 0 |
| Diabetes | 0 | 0 |
| CPD | 4 | 4 |
| PhysiAct | 2 | 2 |
| SES | 3 | 3 |
| PriorHos | 0 | 0 |
| GHQ | 1 | 1 |
| LY | 73.17 | 73.89 |
| Disc LY | 65.37 | 65.79 |
| Disc QALYs | 58.46 | 58.80 |
| Lifetime costs | 338,566.00 | 360,164.58 |
| Disc lifetime costs | 210,573.76 | 225,393.23 |
| ICER per drinker | | 43,923 |

AUDIT: Alcohol Use Disorders Identification Test (score 0–40); BMI: Body Mass Index (0=Normal, 1=Overweight, 2=Obesity); CVD: Cardiovascular Disease; CPD: Number of cigarette per day; PhysiAct: Physical activity (0=No activity, 1=Low, 2=Med, 3=High); SES: Socioeconomic status (1=Most Deprived, 2=2nd, 3=3rd, 4=4th, 5=Least Deprived); PriorHos: Prior Hospitalization (0=No PriorHos, 1=Last Yr, 2=Over last year); GHQ: General Health Questionnaire (1=Best, 2=score 1–3, 3=score 4+). RBT: Random Breath Testing.

1. Male drinkers aged 20 years with harmful alcohol consumption.

|  | Mass media | RBT+Mass media |
| --- | --- | --- |
| Age | 20 | 20 |
| AUDIT | 19 | 19 |
| Binge | 1 | 0 |
| BMI | 0 | 0 |
| CVD_con | 0 | 0 |
| Diabetes | 0 | 0 |
| CPD | 4 | 4 |
| PhysiAct | 2 | 2 |
| SES | 3 | 3 |
| PriorHos | 0 | 0 |
| GHQ | 1 | 1 |
| LY | 71.78 | 73.65 |
| Disc LY | 44.10 | 44.79 |
| Disc QALYs | 39.38 | 39.94 |
| Lifetime costs | 687,453.44 | 737,719.87 |
| Disc lifetime costs | 275,432.97 | 300,648.80 |
| ICER per drinker |  | 44,959 |

AUDIT: Alcohol Use Disorders Identification Test (score 0–40); BMI: Body Mass Index (0=Normal, 1=Overweight, 2=Obesity); CVD: Cardiovascular Disease; CPD: Number of cigarette per day; PhysiAct: Physical activity (0=No activity, 1=Low, 2=Med, 3=High); SES: Socioeconomic status (1=Most Deprived, 2=2nd, 3=3rd, 4=4th, 5=Least Deprived); PriorHos: Prior Hospitalization (0=No PriorHos, 1=Last Yr, 2=Over last year); GHQ: General Health Questionnaire (1=Best, 2=score 1–3, 3=score 4+). RBT: Random Breath Testing.

1. Male drinkers aged 30 years with harmful alcohol consumption.

|  | Mass media | RBT+Mass media |
| --- | --- | --- |
| Age | 30 | 30 |
| AUDIT | 19 | 19 |
| Binge | 1 | 0 |
| BMI | 0 | 0 |
| CVD_con | 0 | 0 |
| Diabetes | 0 | 0 |
| CPD | 4 | 4 |
| PhysiAct | 2 | 2 |
| SES | 3 | 3 |
| PriorHos | 0 | 0 |
| GHQ | 1 | 1 |
| LY | 71.04 | 72.48 |
| Disc LY | 51.53 | 52.16 |
| Disc QALYs | 45.48 | 45.99 |
| Lifetime costs | 564,249.12 | 604,078.62 |
| Disc lifetime costs | 263,505.42 | 285,659.92 |
| ICER per drinker | | 43,502 |

AUDIT: Alcohol Use Disorders Identification Test (score 0–40); BMI: Body Mass Index (0=Normal, 1=Overweight, 2=Obesity); CVD: Cardiovascular Disease; CPD: Number of cigarette per day; PhysiAct: Physical activity (0=No activity, 1=Low, 2=Med, 3=High); SES: Socioeconomic status (1=Most Deprived, 2=2nd, 3=3rd, 4=4th, 5=Least Deprived); PriorHos: Prior Hospitalization (0=No PriorHos, 1=Last Yr, 2=Over last year); GHQ: General Health Questionnaire (1=Best, 2=score 1–3, 3=score 4+). RBT: Random Breath Testing.

1. Male drinkers aged 40 years with harmful alcohol consumption.

|  | Mass media | RBT+Mass media |
| --- | --- | --- |
| Age | 40 | 40 |
| AUDIT | 19 | 19 |
| Binge | 1 | 0 |
| BMI | 0 | 0 |
| CVD_con | 0 | 0 |
| Diabetes | 0 | 0 |
| CPD | 4 | 4 |
| PhysiAct | 2 | 2 |
| SES | 3 | 3 |
| PriorHos | 0 | 0 |
| GHQ | 1 | 1 |
| LY | 71.28 | 72.36 |
| Disc LY | 58.51 | 59.06 |
| Disc QALYs | 51.75 | 52.20 |
| Lifetime costs | 444,601.29 | 475,044.36 |
| Disc lifetime costs | 241,082.25 | 259,821.22 |
| ICER per drinker | | 41,999 |

AUDIT: Alcohol Use Disorders Identification Test (score 0–40); BMI: Body Mass Index (0=Normal, 1=Overweight, 2=Obesity); CVD: Cardiovascular Disease; CPD: Number of cigarette per day; PhysiAct: Physical activity (0=No activity, 1=Low, 2=Med, 3=High); SES: Socioeconomic status (1=Most Deprived, 2=2nd, 3=3rd, 4=4th, 5=Least Deprived); PriorHos: Prior Hospitalization (0=No PriorHos, 1=Last Yr, 2=Over last year); GHQ: General Health Questionnaire (1=Best, 2=score 1–3, 3=score 4+). RBT: Random Breath Testing.

1. Male drinkers aged 50 years with harmful alcohol consumption.

|  | Mass media | RBT+Mass media |
| --- | --- | --- |
| Age | 50 | 50 |
| AUDIT | 19 | 19 |
| Binge | 1 | 0 |
| BMI | 0 | 0 |
| CVD_con | 0 | 0 |
| Diabetes | 0 | 0 |
| CPD | 4 | 4 |
| PhysiAct | 2 | 2 |
| SES | 3 | 3 |
| PriorHos | 0 | 0 |
| GHQ | 1 | 1 |
| LY | 72.90 | 73.68 |
| Disc LY | 65.21 | 65.66 |
| Disc QALYs | 57.28 | 57.65 |
| Lifetime costs | 336,717.47 | 358,844.62 |
| Disc lifetime costs | 210,119.34 | 225,154.97 |
| ICER per drinker | | 41,304 |

AUDIT: Alcohol Use Disorders Identification Test (score 0–40); BMI: Body Mass Index (0=Normal, 1=Overweight, 2=Obesity); CVD: Cardiovascular Disease; CPD: Number of cigarette per day; PhysiAct: Physical activity (0=No activity, 1=Low, 2=Med, 3=High); SES: Socioeconomic status (1=Most Deprived, 2=2nd, 3=3rd, 4=4th, 5=Least Deprived); PriorHos: Prior Hospitalization (0=No PriorHos, 1=Last Yr, 2=Over last year); GHQ: General Health Questionnaire (1=Best, 2=score 1–3, 3=score 4+). RBT: Random Breath Testing.

1. Male drinkers aged 20 years with alcohol dependence.

|  | Mass media | RBT+Mass media |
| --- | --- | --- |
| Age | 20 | 20 |
| AUDIT | 31 | 31 |
| Binge | 1 | 0 |
| BMI | 0 | 0 |
| CVD_con | 0 | 0 |
| Diabetes | 0 | 0 |
| CPD | 4 | 4 |
| PhysiAct | 2 | 2 |
| SES | 3 | 3 |
| PriorHos | 0 | 0 |
| GHQ | 1 | 1 |
| LY | 70.21 | 72.59 |
| Disc LY | 43.39 | 44.29 |
| Disc QALYs | 36.70 | 37.41 |
| Lifetime costs | 677,004.94 | 731,842.22 |
| Disc lifetime costs | 274,970.35 | 301,310.18 |
| ICER per drinker | | 37,093 |

AUDIT: Alcohol Use Disorders Identification Test (score 0–40); BMI: Body Mass Index (0=Normal, 1=Overweight, 2=Obesity); CVD: Cardiovascular Disease; CPD: Number of cigarette per day; PhysiAct: Physical activity (0=No activity, 1=Low, 2=Med, 3=High); SES: Socioeconomic status (1=Most Deprived, 2=2nd, 3=3rd, 4=4th, 5=Least Deprived); PriorHos: Prior Hospitalization (0=No PriorHos, 1=Last Yr, 2=Over last year); GHQ: General Health Questionnaire (1=Best, 2=score 1–3, 3=score 4+). RBT: Random Breath Testing.

1. Male drinkers aged 30 years with alcohol dependence.

|  | Mass media | RBT+Mass media |
| --- | --- | --- |
| Age | 30 | 30 |
| AUDIT | 31 | 31 |
| Binge | 1 | 0 |
| BMI | 0 | 0 |
| CVD_con | 0 | 0 |
| Diabetes | 0 | 0 |
| CPD | 4 | 4 |
| PhysiAct | 2 | 2 |
| SES | 3 | 3 |
| PriorHos | 0 | 0 |
| GHQ | 1 | 1 |
| LY | 69.66 | 71.52 |
| Disc LY | 50.84 | 51.68 |
| Disc QALYs | 42.47 | 43.12 |
| Lifetime costs | 554,162.14 | 597,768.83 |
| Disc lifetime costs | 262,009.87 | 285,330.02 |
| ICER per drinker |  | 35,890 |

AUDIT: Alcohol Use Disorders Identification Test (score 0–40); BMI: Body Mass Index (0=Normal, 1=Overweight, 2=Obesity); CVD: Cardiovascular Disease; CPD: Number of cigarette per day; PhysiAct: Physical activity (0=No activity, 1=Low, 2=Med, 3=High); SES: Socioeconomic status (1=Most Deprived, 2=2nd, 3=3rd, 4=4th, 5=Least Deprived); PriorHos: Prior Hospitalization (0=No PriorHos, 1=Last Yr, 2=Over last year); GHQ: General Health Questionnaire (1=Best, 2=score 1–3, 3=score 4+). RBT: Random Breath Testing.

1. Male drinkers aged 40 years with alcohol dependence.

|  | Mass media | RBT+Mass media |
| --- | --- | --- |
| Age | 40 | 40 |
| AUDIT | 31 | 31 |
| Binge | 1 | 0 |
| BMI | 0 | 0 |
| CVD_con | 0 | 0 |
| Diabetes | 0 | 0 |
| CPD | 4 | 4 |
| PhysiAct | 2 | 2 |
| SES | 3 | 3 |
| PriorHos | 0 | 0 |
| GHQ | 1 | 1 |
| LY | 70.12 | 71.53 |
| Disc LY | 57.87 | 58.60 |
| Disc QALYs | 48.44 | 49.01 |
| Lifetime costs | 435,702.78 | 469,038.25 |
| Disc lifetime costs | 238,984.42 | 258,809.12 |
| ICER per drinker | | 34,975 |

AUDIT: Alcohol Use Disorders Identification Test (score 0–40); BMI: Body Mass Index (0=Normal, 1=Overweight, 2=Obesity); CVD: Cardiovascular Disease; CPD: Number of cigarette per day; PhysiAct: Physical activity (0=No activity, 1=Low, 2=Med, 3=High); SES: Socioeconomic status (1=Most Deprived, 2=2nd, 3=3rd, 4=4th, 5=Least Deprived); PriorHos: Prior Hospitalization (0=No PriorHos, 1=Last Yr, 2=Over last year); GHQ: General Health Questionnaire (1=Best, 2=score 1–3, 3=score 4+). RBT: Random Breath Testing.

1. Male drinkers aged 50 years with alcohol dependence.

|  | Mass media | RBT+Mass media |
| --- | --- | --- |
| Age | 50 | 50 |
| AUDIT | 31 | 31 |
| Binge | 1 | 0 |
| BMI | 0 | 0 |
| CVD_con | 0 | 0 |
| Diabetes | 0 | 0 |
| CPD | 4 | 4 |
| PhysiAct | 2 | 2 |
| SES | 3 | 3 |
| PriorHos | 0 | 0 |
| GHQ | 1 | 1 |
| LY | 71.98 | 72.97 |
| Disc LY | 64.64 | 65.23 |
| Disc QALYs | 53.72 | 54.17 |
| Lifetime costs | 329,654.55 | 353,719.31 |
| Disc lifetime costs | 207,931.97 | 223,824.03 |
| ICER per drinker |  | 35,232 |

AUDIT: Alcohol Use Disorders Identification Test (score 0–40); BMI: Body Mass Index (0=Normal, 1=Overweight, 2=Obesity); CVD: Cardiovascular Disease; CPD: Number of cigarette per day; PhysiAct: Physical activity (0=No activity, 1=Low, 2=Med, 3=High); SES: Socioeconomic status (1=Most Deprived, 2=2nd, 3=3rd, 4=4th, 5=Least Deprived); PriorHos: Prior Hospitalization (0=No PriorHos, 1=Last Yr, 2=Over last year); GHQ: General Health Questionnaire (1=Best, 2=score 1–3, 3=score 4+). RBT: Random Breath Testing.

**Appendix 2: Sub-group analyses for female drinkers**

1. Female drinkers aged 30 years old with low levels of alcohol consumption.

|  | Mass media | RBT+Mass media |
| --- | --- | --- |
| Age | 30 | 30 |
| AUDIT | 7 | 7 |
| Binge | 1 | 0 |
| BMI | 0 | 0 |
| CVD_con | 0 | 0 |
| Diabetes | 0 | 0 |
| CPD | 4 | 4 |
| PhysiAct | 2 | 2 |
| SES | 3 | 3 |
| PriorHos | 0 | 0 |
| GHQ | 1 | 1 |
| LY | 72.70 | 73.15 |
| Disc LY | 51.04 | 51.19 |
| Disc QALYs | 39.91 | 40.01 |
| Lifetime costs | 550,153.75 | 564,655.88 |
| Disc lifetime costs | 252,881.24 | 262,370.06 |
| ICER per drinker |  | 98,243 |

AUDIT: Alcohol Use Disorders Identification Test (score 0–40); BMI: Body Mass Index (0=Normal, 1=Overweight, 2=Obesity); CVD: Cardiovascular Disease; CPD: Number of cigarette per day; PhysiAct: Physical activity (0=No activity, 1=Low, 2=Med, 3=High); SES: Socioeconomic status (1=Most Deprived, 2=2nd, 3=3rd, 4=4th, 5=Least Deprived); PriorHos: Prior Hospitalization (0=No PriorHos, 1=Last Yr, 2=Over last year); GHQ: General Health Questionnaire (1=Best, 2=score 1–3, 3=score 4+). RBT: Random Breath Testing.

1. Female drinkers aged 40 years with low alcohol consumption.

|  | Mass media | RBT+Mass media |
| --- | --- | --- |
| Age | 40 | 40 |
| AUDIT | 7 | 7 |
| Binge | 1 | 0 |
| BMI | 0 | 0 |
| CVD_con | 0 | 0 |
| Diabetes | 0 | 0 |
| CPD | 4 | 4 |
| PhysiAct | 2 | 2 |
| SES | 3 | 3 |
| PriorHos | 0 | 0 |
| GHQ | 1 | 1 |
| LY | 70.35 | 70.66 |
| Disc LY | 57.50 | 57.63 |
| QALY | 44.00 | 44.08 |
| Disc QALYs | 403,316.62 | 414,916.20 |
| Lifetime costs | 220,228.77 | 228,609.74 |
| ICER per drinker |  | 101,970 |

AUDIT: Alcohol Use Disorders Identification Test (score 0–40); BMI: Body Mass Index (0=Normal, 1=Overweight, 2=Obesity); CVD: Cardiovascular Disease; CPD: Number of cigarette per day; PhysiAct: Physical activity (0=No activity, 1=Low, 2=Med, 3=High); SES: Socioeconomic status (1=Most Deprived, 2=2nd, 3=3rd, 4=4th, 5=Least Deprived); PriorHos: Prior Hospitalization (0=No PriorHos, 1=Last Yr, 2=Over last year); GHQ: General Health Questionnaire (1=Best, 2=score 1–3, 3=score 4+). RBT: Random Breath Testing.

1. Female drinkers aged 50 years with low alcohol consumption.

|  | Mass media | RBT+Mass media |
| --- | --- | --- |
| Age | 50 | 50 |
| AUDIT | 7 | 7 |
| Binge | 1 | 0 |
| BMI | 0 | 0 |
| CVD_con | 0 | 0 |
| Diabetes | 0 | 0 |
| CPD | 4 | 4 |
| PhysiAct | 2 | 2 |
| SES | 3 | 3 |
| PriorHos | 0 | 0 |
| GHQ | 1 | 1 |
| LY | 70.86 | 71.05 |
| Disc LY | 63.86 | 63.96 |
| Disc QALYs | 48.34 | 48.40 |
| Lifetime costs | 285,748.08 | 294,766.88 |
| Disc lifetime costs | 182,538.53 | 189,717.54 |
| ICER per drinker | | 119,948 |

AUDIT: Alcohol Use Disorders Identification Test (score 0–40); BMI: Body Mass Index (0=Normal, 1=Overweight, 2=Obesity); CVD: Cardiovascular Disease; CPD: Number of cigarette per day; PhysiAct: Physical activity (0=No activity, 1=Low, 2=Med, 3=High); SES: Socioeconomic status (1=Most Deprived, 2=2nd, 3=3rd, 4=4th, 5=Least Deprived); PriorHos: Prior Hospitalization (0=No PriorHos, 1=Last Yr, 2=Over last year); GHQ: General Health Questionnaire (1=Best, 2=score 1–3, 3=score 4+). RBT: Random Breath Testing.

1. Female drinkers aged 20 years with hazardous alcohol consumption.

|  | Mass media | RBT+Mass media |
| --- | --- | --- |
| Age | 20 | 20 |
| AUDIT | 15 | 15 |
| Binge | 1 | 0 |
| BMI | 0 | 0 |
| CVD_con | 0 | 0 |
| Diabetes | 0 | 0 |
| CPD | 4 | 4 |
| PhysiAct | 2 | 2 |
| SES | 3 | 3 |
| PriorHos | 0 | 0 |
| GHQ | 1 | 1 |
| LY | 76.46 | 77.16 |
| Disc LY | 43.89 | 44.10 |
| Disc QALYs | 32.42 | 32.55 |
| Lifetime costs | 703,796.18 | 722,267.47 |
| Disc lifetime costs | 273,805.65 | 284,602.59 |
| ICER per drinker |  | 80,964 |

AUDIT: Alcohol Use Disorders Identification Test (score 0–40); BMI: Body Mass Index (0=Normal, 1=Overweight, 2=Obesity); CVD: Cardiovascular Disease; CPD: Number of cigarette per day; PhysiAct: Physical activity (0=No activity, 1=Low, 2=Med, 3=High); SES: Socioeconomic status (1=Most Deprived, 2=2nd, 3=3rd, 4=4th, 5=Least Deprived); PriorHos: Prior Hospitalization (0=No PriorHos, 1=Last Yr, 2=Over last year); GHQ: General Health Questionnaire (1=Best, 2=score 1–3, 3=score 4+). RBT: Random Breath Testing.

1. Female drinkers aged 30 years with hazardous alcohol consumption.

|  | Mass media | RBT+Mass media |
| --- | --- | --- |
| Age | 30 | 30 |
| AUDIT | 15 | 15 |
| Binge | 1 | 0 |
| BMI | 0 | 0 |
| CVD_con | 0 | 0 |
| Diabetes | 0 | 0 |
| CPD | 4 | 4 |
| PhysiAct | 2 | 2 |
| SES | 3 | 3 |
| PriorHos | 0 | 0 |
| GHQ | 1 | 1 |
| LY | 72.00 | 72.53 |
| Disc LY | 50.74 | 50.93 |
| Disc QALYs | 38.72 | 38.84 |
| Lifetime costs | 541,541.17 | 556,863.84 |
| Disc lifetime costs | 249,659.37 | 259,450.81 |
| ICER per drinker | | 82,697 |

AUDIT: Alcohol Use Disorders Identification Test (score 0–40); BMI: Body Mass Index (0=Normal, 1=Overweight, 2=Obesity); CVD: Cardiovascular Disease; CPD: Number of cigarette per day; PhysiAct: Physical activity (0=No activity, 1=Low, 2=Med, 3=High); SES: Socioeconomic status (1=Most Deprived, 2=2nd, 3=3rd, 4=4th, 5=Least Deprived); PriorHos: Prior Hospitalization (0=No PriorHos, 1=Last Yr, 2=Over last year); GHQ: General Health Questionnaire (1=Best, 2=score 1–3, 3=score 4+). RBT: Random Breath Testing.

1. Female drinkers aged 40 years with hazardous alcohol consumption.

|  | Mass media | RBT+Mass media |
| --- | --- | --- |
| Age | 40 | 40 |
| AUDIT | 15 | 15 |
| Binge | 1 | 0 |
| BMI | 0 | 0 |
| CVD_con | 0 | 0 |
| Diabetes | 0 | 0 |
| CPD | 4 | 4 |
| PhysiAct | 2 | 2 |
| SES | 3 | 3 |
| PriorHos | 0 | 0 |
| GHQ | 1 | 1 |
| LY | 69.78 | 70.16 |
| Disc LY | 57.20 | 57.37 |
| Disc QALYs | 42.70 | 42.80 |
| Lifetime costs | 396,245.92 | 408,535.32 |
| Disc lifetime costs | 217,035.64 | 225,721.89 |
| ICER per drinker | | 83,448 |

AUDIT: Alcohol Use Disorders Identification Test (score 0–40); BMI: Body Mass Index (0=Normal, 1=Overweight, 2=Obesity); CVD: Cardiovascular Disease; CPD: Number of cigarette per day; PhysiAct: Physical activity (0=No activity, 1=Low, 2=Med, 3=High); SES: Socioeconomic status (1=Most Deprived, 2=2nd, 3=3rd, 4=4th, 5=Least Deprived); PriorHos: Prior Hospitalization (0=No PriorHos, 1=Last Yr, 2=Over last year); GHQ: General Health Questionnaire (1=Best, 2=score 1–3, 3=score 4+). RBT: Random Breath Testing.

1. Female drinkers aged 50 years with hazardous alcohol consumption.

|  | Mass media | RBT+Mass media |
| --- | --- | --- |
| Age | 50 | 50 |
| AUDIT | 15 | 15 |
| Binge | 1 | 0 |
| BMI | 0 | 0 |
| CVD_con | 0 | 0 |
| Diabetes | 0 | 0 |
| CPD | 4 | 4 |
| PhysiAct | 2 | 2 |
| SES | 3 | 3 |
| PriorHos | 0 | 0 |
| GHQ | 1 | 1 |
| LY | 70.43 | 70.68 |
| Disc LY | 63.61 | 63.74 |
| Disc QALYs | 46.95 | 47.03 |
| Lifetime costs | 280,372.58 | 289,940.18 |
| Disc lifetime costs | 179,658.04 | 187,121.54 |
| ICER per drinker | | 92,109 |

AUDIT: Alcohol Use Disorders Identification Test (score 0–40); BMI: Body Mass Index (0=Normal, 1=Overweight, 2=Obesity); CVD: Cardiovascular Disease; CPD: Number of cigarette per day; PhysiAct: Physical activity (0=No activity, 1=Low, 2=Med, 3=High); SES: Socioeconomic status (1=Most Deprived, 2=2nd, 3=3rd, 4=4th, 5=Least Deprived); PriorHos: Prior Hospitalization (0=No PriorHos, 1=Last Yr, 2=Over last year); GHQ: General Health Questionnaire (1=Best, 2=score 1–3, 3=score 4+). RBT: Random Breath Testing.

1. Female drinkers aged 20 years with harmful alcohol consumption.

|  | Mass media | RBT+Mass media |
| --- | --- | --- |
| Age | 20 | 20 |
| AUDIT | 19 | 19 |
| Binge | 1 | 0 |
| BMI | 0 | 0 |
| CVD_con | 0 | 0 |
| Diabetes | 0 | 0 |
| CPD | 4 | 4 |
| PhysiAct | 2 | 2 |
| SES | 3 | 3 |
| PriorHos | 0 | 0 |
| GHQ | 1 | 1 |
| LY | 76.01 | 76.73 |
| Disc LY | 43.73 | 43.94 |
| Disc QALYs | 31.88 | 32.02 |
| Lifetime costs | 698,248.39 | 717,085.30 |
| Disc lifetime costs | 272,116.97 | 283,019.35 |
| ICER per drinker |  | 80,953 |

AUDIT: Alcohol Use Disorders Identification Test (score 0–40); BMI: Body Mass Index (0=Normal, 1=Overweight, 2=Obesity); CVD: Cardiovascular Disease; CPD: Number of cigarette per day; PhysiAct: Physical activity (0=No activity, 1=Low, 2=Med, 3=High); SES: Socioeconomic status (1=Most Deprived, 2=2nd, 3=3rd, 4=4th, 5=Least Deprived); PriorHos: Prior Hospitalization (0=No PriorHos, 1=Last Yr, 2=Over last year); GHQ: General Health Questionnaire (1=Best, 2=score 1–3, 3=score 4+). RBT: Random Breath Testing.

1. Female drinkers aged 30 years with harmful alcohol consumption.

|  | Mass media | RBT+Mass media |
| --- | --- | --- |
| Age | 30 | 30 |
| AUDIT | 19 | 19 |
| Binge | 1 | 0 |
| BMI | 0 | 0 |
| CVD_con | 0 | 0 |
| Diabetes | 0 | 0 |
| CPD | 4 | 4 |
| PhysiAct | 2 | 2 |
| SES | 3 | 3 |
| PriorHos | 0 | 0 |
| GHQ | 1 | 1 |
| LY | 71.62 | 72.18 |
| Disc LY | 50.57 | 50.78 |
| Disc QALYs | 38.12 | 38.25 |
| Lifetime costs | 536,784.63 | 552,544.08 |
| Disc lifetime costs | 247,901.92 | 257,848.75 |
| ICER per drinker | | 76,196 |

AUDIT: Alcohol Use Disorders Identification Test (score 0–40); BMI: Body Mass Index (0=Normal, 1=Overweight, 2=Obesity); CVD: Cardiovascular Disease; CPD: Number of cigarette per day; PhysiAct: Physical activity (0=No activity, 1=Low, 2=Med, 3=High); SES: Socioeconomic status (1=Most Deprived, 2=2nd, 3=3rd, 4=4th, 5=Least Deprived); PriorHos: Prior Hospitalization (0=No PriorHos, 1=Last Yr, 2=Over last year); GHQ: General Health Questionnaire (1=Best, 2=score 1–3, 3=score 4+). RBT: Random Breath Testing.

1. Female drinkers aged 40 years with harmful alcohol consumption.

|  | Mass media | RBT+Mass media |
| --- | --- | --- |
| Age | 40 | 40 |
| AUDIT | 19 | 19 |
| Binge | 1 | 0 |
| BMI | 0 | 0 |
| CVD_con | 0 | 0 |
| Diabetes | 0 | 0 |
| CPD | 4 | 4 |
| PhysiAct | 2 | 2 |
| SES | 3 | 3 |
| PriorHos | 0 | 0 |
| GHQ | 1 | 1 |
| LY | 69.47 | 69.88 |
| Disc LY | 57.04 | 57.23 |
| Disc QALYs | 42.04 | 42.15 |
| Lifetime costs | 392,387.55 | 405,037.48 |
| Disc lifetime costs | 215,316.59 | 224,156.87 |
| ICER per drinker | | 76,190 |

AUDIT: Alcohol Use Disorders Identification Test (score 0–40); BMI: Body Mass Index (0=Normal, 1=Overweight, 2=Obesity); CVD: Cardiovascular Disease; CPD: Number of cigarette per day; PhysiAct: Physical activity (0=No activity, 1=Low, 2=Med, 3=High); SES: Socioeconomic status (1=Most Deprived, 2=2nd, 3=3rd, 4=4th, 5=Least Deprived); PriorHos: Prior Hospitalization (0=No PriorHos, 1=Last Yr, 2=Over last year); GHQ: General Health Questionnaire (1=Best, 2=score 1–3, 3=score 4+). RBT: Random Breath Testing.

1. Female drinkers aged 50 years with harmful alcohol consumption.

|  | Mass media | RBT+Mass media |
| --- | --- | --- |
| Age | 50 | 50 |
| AUDIT | 19 | 19 |
| Binge | 1 | 0 |
| BMI | 0 | 0 |
| CVD_con | 0 | 0 |
| Diabetes | 0 | 0 |
| CPD | 4 | 4 |
| PhysiAct | 2 | 2 |
| SES | 3 | 3 |
| PriorHos | 0 | 0 |
| GHQ | 1 | 1 |
| LY | 70.16 | 70.43 |
| Disc LY | 63.44 | 63.59 |
| Disc QALYs | 46.22 | 46.31 |
| Lifetime costs | 277,262.52 | 287,113.13 |
| Disc lifetime costs | 178,021.79 | 185,626.98 |
| ICER per drinker | | 81,873 |

AUDIT: Alcohol Use Disorders Identification Test (score 0–40); BMI: Body Mass Index (0=Normal, 1=Overweight, 2=Obesity); CVD: Cardiovascular Disease; CPD: Number of cigarette per day; PhysiAct: Physical activity (0=No activity, 1=Low, 2=Med, 3=High); SES: Socioeconomic status (1=Most Deprived, 2=2nd, 3=3rd, 4=4th, 5=Least Deprived); PriorHos: Prior Hospitalization (0=No PriorHos, 1=Last Yr, 2=Over last year); GHQ: General Health Questionnaire (1=Best, 2=score 1–3, 3=score 4+). RBT: Random Breath Testing.

1. Female drinkers aged 20 years with alcohol dependence.

|  | Mass media | RBT+Mass media |
| --- | --- | --- |
| Age | 20 | 20 |
| AUDIT | 31 | 31 |
| Binge | 1 | 0 |
| BMI | 0 | 0 |
| CVD_con | 0 | 0 |
| Diabetes | 0 | 0 |
| CPD | 4 | 4 |
| PhysiAct | 2 | 2 |
| SES | 3 | 3 |
| PriorHos | 0 | 0 |
| GHQ | 1 | 1 |
| LY | 74.30 | 75.18 |
| Disc LY | 43.08 | 43.37 |
| Disc QALYs | 30.18 | 30.36 |
| Lifetime costs | 678,025.95 | 698,524.79 |
| Disc lifetime costs | 266,017.48 | 277,386.93 |
| ICER per drinker | | 63,870 |

AUDIT: Alcohol Use Disorders Identification Test (score 0–40); BMI: Body Mass Index (0=Normal, 1=Overweight, 2=Obesity); CVD: Cardiovascular Disease; CPD: Number of cigarette per day; PhysiAct: Physical activity (0=No activity, 1=Low, 2=Med, 3=High); SES: Socioeconomic status (1=Most Deprived, 2=2nd, 3=3rd, 4=4th, 5=Least Deprived); PriorHos: Prior Hospitalization (0=No PriorHos, 1=Last Yr, 2=Over last year); GHQ: General Health Questionnaire (1=Best, 2=score 1–3, 3=score 4+). RBT: Random Breath Testing.

1. Female drinkers aged 30 years with alcohol dependence.

|  | Mass media | RBT+Mass media |
| --- | --- | --- |
| Age | 30 | 30 |
| AUDIT | 31 | 31 |
| Binge | 1 | 0 |
| BMI | 0 | 0 |
| CVD_con | 0 | 0 |
| Diabetes | 0 | 0 |
| CPD | 4 | 4 |
| PhysiAct | 2 | 2 |
| SES | 3 | 3 |
| PriorHos | 0 | 0 |
| GHQ | 1 | 1 |
| LY | 70.15 | 70.86 |
| Disc LY | 49.92 | 50.19 |
| Disc QALYs | 36.22 | 36.39 |
| Lifetime costs | 519,512.98 | 536,703.35 |
| Disc lifetime costs | 241,575.56 | 252,013.39 |
| ICER per drinker |  | 60,297 |

AUDIT: Alcohol Use Disorders Identification Test (score 0–40); BMI: Body Mass Index (0=Normal, 1=Overweight, 2=Obesity); CVD: Cardiovascular Disease; CPD: Number of cigarette per day; PhysiAct: Physical activity (0=No activity, 1=Low, 2=Med, 3=High); SES: Socioeconomic status (1=Most Deprived, 2=2nd, 3=3rd, 4=4th, 5=Least Deprived); PriorHos: Prior Hospitalization (0=No PriorHos, 1=Last Yr, 2=Over last year); GHQ: General Health Questionnaire (1=Best, 2=score 1–3, 3=score 4+). RBT: Random Breath Testing.

1. Female drinkers aged 40 years with alcohol dependence.

|  | Mass media | RBT+Mass media |
| --- | --- | --- |
| Age | 40 | 40 |
| AUDIT | 31 | 31 |
| Binge | 1 | 0 |
| BMI | 0 | 0 |
| CVD_con | 0 | 0 |
| Diabetes | 0 | 0 |
| CPD | 4 | 4 |
| PhysiAct | 2 | 2 |
| SES | 3 | 3 |
| PriorHos | 0 | 0 |
| GHQ | 1 | 1 |
| LY | 68.26 | 68.79 |
| Disc LY | 56.40 | 56.65 |
| Disc QALYs | 39.96 | 40.12 |
| Lifetime costs | 378,404.60 | 392,207.50 |
| Disc lifetime costs | 209,150.35 | 218,465.58 |
| ICER per drinker | | 59,384 |

AUDIT: Alcohol Use Disorders Identification Test (score 0–40); BMI: Body Mass Index (0=Normal, 1=Overweight, 2=Obesity); CVD: Cardiovascular Disease; CPD: Number of cigarette per day; PhysiAct: Physical activity (0=No activity, 1=Low, 2=Med, 3=High); SES: Socioeconomic status (1=Most Deprived, 2=2nd, 3=3rd, 4=4th, 5=Least Deprived); PriorHos: Prior Hospitalization (0=No PriorHos, 1=Last Yr, 2=Over last year); GHQ: General Health Questionnaire (1=Best, 2=score 1–3, 3=score 4+). RBT: Random Breath Testing.

1. Female drinkers aged 50 years with alcohol dependence.

|  | Mass media | RBT+Mass media |
| --- | --- | --- |
| Age | 50 | 50 |
| AUDIT | 31 | 31 |
| Binge | 1 | 0 |
| BMI | 0 | 0 |
| CVD_con | 0 | 0 |
| Diabetes | 0 | 0 |
| CPD | 4 | 4 |
| PhysiAct | 2 | 2 |
| SES | 3 | 3 |
| PriorHos | 0 | 0 |
| GHQ | 1 | 1 |
| LY | 69.23 | 69.60 |
| Disc LY | 62.87 | 63.08 |
| Disc QALYs | 44.01 | 44.14 |
| Lifetime costs | 266,705.96 | 277,432.96 |
| Disc lifetime costs | 172,540.79 | 180,566.16 |
| ICER per drinker |  | 60,998 |

AUDIT: Alcohol Use Disorders Identification Test (score 0–40); BMI: Body Mass Index (0=Normal, 1=Overweight, 2=Obesity); CVD: Cardiovascular Disease; CPD: Number of cigarette per day; PhysiAct: Physical activity (0=No activity, 1=Low, 2=Med, 3=High); SES: Socioeconomic status (1=Most Deprived, 2=2nd, 3=3rd, 4=4th, 5=Least Deprived); PriorHos: Prior Hospitalization (0=No PriorHos, 1=Last Yr, 2=Over last year); GHQ: General Health Questionnaire (1=Best, 2=score 1–3, 3=score 4+). RBT: Random Breath Testing.
